# ERK signalling modulates epigenome to drive epithelial to mesenchymal transition

**DOI:** 10.18632/oncotarget.16493

**Published:** 2017-03-23

**Authors:** Mohit Navandar, Angela Garding, Sanjeeb Kumar Sahu, Abhijeet Pataskar, Sandra Schick, Vijay K. Tiwari

**Affiliations:** ^1^ Institute of Molecular Biology (IMB), Mainz, Germany

**Keywords:** EMT, ERK, epigenetics, transcription factors, gene regulation

## Abstract

The series of events that allow the conversion from adherent epithelial cells into migratory cells is collectively known as epithelial-mesenchymal transition (EMT). EMT is employed during embryonic development such as for gastrulation and neural crest migration and is misused in diseases, such as cancer metastasis. ERK signalling is known to be essential for EMT, however its influence on the epigenetic and transcriptional programme underlying EMT is poorly understood. Here, using a comprehensive genome-wide analysis of H3K27ac mark and gene expression in mammary epithelial cells undergoing EMT, we found that ERK signalling is essential for the epigenetic reprogramming underlying hallmark gene expression and phenotypic changes of EMT. We show that the chemical inhibition of Erk signalling during EMT prevents the loss and gain of the H3K27ac mark at regulatory regions of epithelial and mesenchymal genes, respectively, and results in a transcriptome and epigenome closer to those of epithelial cells. Further computational analyses identified a distinct set of transcription factor motifs enriched at distal regulatory regions that are epigenetically remodelled by ERK signalling. Altogether, our findings reveal an ERK-dependent epigenetic remodelling of regulatory elements that results in a gene expression programme essential for driving EMT.

## INTRODUCTION

Epithelial to mesenchymal transition (EMT) is a process of phenotypic remodelling that enables cells to detach from their surroundings by losing cell-to-cell connections and to subsequently migrate. Such an acquisition of a motile, mesenchymal phenotype involves cytoskeletal remodelling, changing of cellular shape and the resolution of cellular adhesion [1, 2]. EMT occurs primarily during embryonic development [1], tissue regeneration and wound healing [3, 4], and is also misused in disease conditions, such as fibrosis [5] and metastasis formation during tumorigenesis [6]. This process is primarily induced by cytokines, such as transforming growth factor β (TGF-β), which are secreted by the cells and stimulate several signalling cascades that result in phenotypic remodelling. The RAS–RAF–MEK–ERK MAPK signalling cascade is one of such cascades that is known to be essential for the acquisition of a mesenchymal phenotype [7–9]. However, very little is known regarding the mechanism of ERK signalling action during EMT. Importantly, it is yet elusive how ERK signalling modulates EMT gene expression program and whether this involves modulation of the epigenome.

It is increasingly appreciated that a variety of signalling cascades involve remodelling of the chromatin landscape for the consequent translation of extracellular cues into the transcriptional response of the cell [10]. Such rearrangement of the epigenome may be achieved by signalling pathways targeting chromatin modifying enzymes, transcription factors and histone chaperones. In line with this, genome-wide analysis of chromatin marks during TGF-β-induced EMT previously revealed a reorganization of specific chromatin domains in this process that involved an LSD1-dependent reduction of heterochromatic marks and an increase in euchromatic marks [11]. Interestingly, ERK was recently shown to phosphorylate MCRIP1, which in turn releases the transcriptional co-repressor CTBP to interact with Zeb1 at target gene promoters, such as E-cadherin, and represses transcription [12]. Furthermore, ERK is thought to modify chromatin by directly phosphorylating histones or inducing downstream factors such as MSK1, AuroraB and PKM2 that have been shown to modify histone 3 at serine 10 (H3S10) [13] [14] [15]. Moreover, there are also reports of a direct binding of ERK to chromatin in mouse and human embryonic stem cells [16] [17, 18]. However it is not explored whether ERK functions similarly via epigenome modulation during EMT. Therefore, we sought to elucidate the effects of ERK signalling cascade on chromatin and transcriptome landscape during EMT.

Here, we found that cells deficient in ERK signalling fail to undergo proper EMT. Using global gene expression profiling, we identified a significant set of epithelial and mesenchymal genes under the direct transcriptional control of the ERK signalling pathway. Importantly, we show that a large fraction of these gene expression changes can be explained by ERK-dependent epigenetic remodelling of their regulatory elements. These regulatory regions are enriched for distinct transcription factor motifs and provide candidate downstream effectors of the ERK pathway for further investigation.

## RESULTS

### ERK signalling is required for phenotypic remodelling during TGF-β-induced EMT

**Figure 1:**
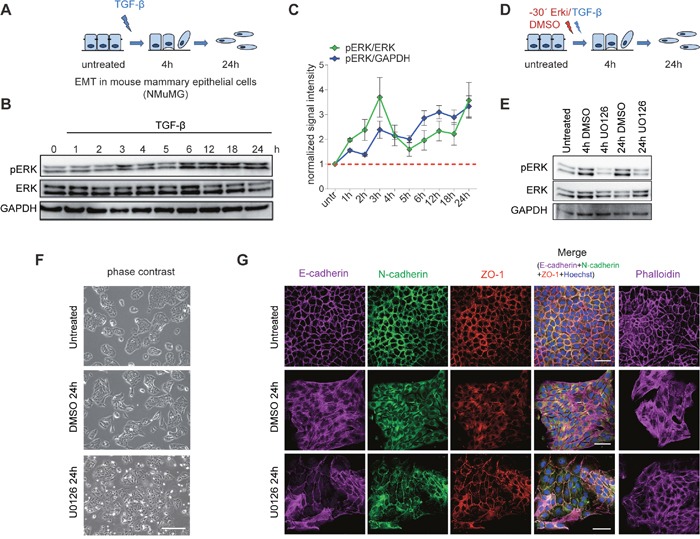
Inhibition of ERK signalling impairs EMT **(A)** EMT in mouse mammary epithelial cells (NMuMG) upon TGFβ treatment. **(B)** Representative immunoblots showing the phosphorylation state of ERK during TGF-β-induced EMT in NMuMG cells. GAPDH and total EKR are loading controls. **(C)** Quantification of pERK signal intensity with respect to total ERK (green line) and with respect to GAPDH (blue line) using ImageJ software. Fold change with respect to untreated time point is plotted on the y-axis and the phosphorylation state at untreated state is indicated by the red dotted line. **(D)** Graphical representation of the treatment scheme used for the inhibition of ERK signalling pathway during EMT. NMuMG cells were treated with an ERK inhibitor (UO126) 30 minutes before TGF-β stimulation. **(E)** Representative immunoblots showing the phosphorylation state of ERK at 4h and 24h of TGF-β-induced EMT with (UO126) or without (DMSO) ERK inhibition in NMuMG cells. GAPDH and total ERK serve loading controls. **(F)** Phase contrast images of untreated NMuMG cells and cells treated for 24h with TGF-β in the presence (UO126) or absence (DMSO) of the ERK inhibitor. **(G)** Immunofluorescence microscopy analysis of changes in the localization and expression levels of marker proteins in cells treated as in **(F)**. The staining was performed with antibodies against the epithelial markers E-cadherin and ZO-1 and the mesenchymal markers N-cadherin and with phalloidin to visualize the actin cytoskeleton. Scale bar, 100 μm, 63X magnification.

To address the dynamics of ERK signalling during EMT, we used untransformed normal murine mammary gland (NMuMG) epithelial cells exposed to TGF-β, which is an established model for EMT in cell culture [19, 20] (Figure 1A). Using this system, we first assessed the phosphorylation kinetics of the kinases Mapk1 (ERK2) and Mapk3 (ERK1) during TGF-β-induced EMT. An immunoblot analysis of the phosphorylated ERK isoforms during an EMT time-course experiment showed that while ERK phosphorylation was upregulated within 1 hour of EMT initiation, its first strong induction was observed at the 3 hours time point, which continued to show gradual increase until 24 hours (Figure 1B and 1C). Guided by this activation pattern of ERK signalling, we chemically inhibited the pathway at both an early (4h) and late (24h) time point of TGF-β-induced EMT. Following a defined treatment scheme using an established inhibitor of ERK signalling, UO126, 30 minutes before TGF-β induction (Figure 1D), we observed a significant blockage of ERK activation both at 4h and 24h (Figure 1E). The inhibition of the ERK signalling cascade perturbed the acquisition of the phenotypic changes observed after 24h of TGF-β induction (Figure 1F and Supplementary Figure 1A). Overall, these cells were less elongated, did not resolve cell-to-cell connections and appeared more similar to the untreated epithelial control cells (Figure 1F and Supplementary Figure 1A). This phenotypic observation from bright field images was strongly supported by immunofluorescence staining for key EMT markers (Figure 1G). The cell junction markers, including ZO-1, the epithelial cadherin Cdh1 as well as the mesenchymal cadherin Cdh2 lost their membrane-associated localization during EMT and displayed a more dispersed staining in the entire cytosol during EMT. However, they remained localized to the membrane in the cells treated with the ERK inhibitor, thus suggesting that the cells retained an epithelial phenotype (Figure 1G). These observations were further validated using an independent ERK inhibitor PD98095 (Supplementary Figure 1A and 1B). Furthermore, F-actin, which shows a distinct staining of the cellular membrane in untreated cells, forms stress fibres in the mesenchymal cells but stays membrane-associated after the inhibition of ERK. Together, our findings indicate that ERK signalling is required for TGF-β-induced EMT, a conclusion in line with previous observations [7, 8].

### Inhibition of ERK signalling results in an aberrant EMT gene expression program

**Figure 2:**
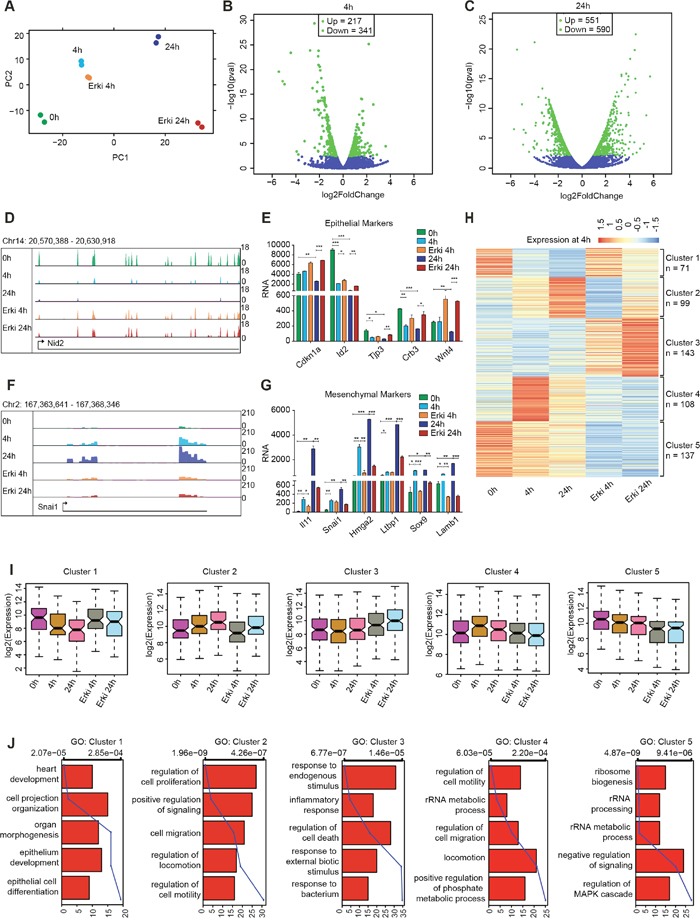
ERK signalling is essential for gene expression reprogramming during EMT **(A)** Scatter plot representing the variation contributed by the first two components from a principal component analysis performed on the transcriptomes from DMSO EMT time points (0h, 4h and 24h) and after ERK inhibition (ERKi 4h and ERKi 24h). The first two principal components (PCs) are represented as distance on the x- and y-axis and represent the variation between the samples. **(B-C)** Volcano plots representing the differential expression of genes in 4h DMSO and 4h ERKi conditions **(B)** and 24h DMSO and 24h ERKi conditions **(C)**. **(D)** UCSC Genome Browser track expression plot of the epithelial gene Nid2 for all afore mentioned conditions. **(E)** Bar plot depicting the expression of epithelial marker genes during all time points of DMSO EMT and after ERK inhibition. Significance was calculated using T-Test (* p<0.05; ** p<0.01; ***<0.001). **(F-G)** The same as **(D)** and **(E)** but for mesenchymal genes. **(H)** Heatmap representation at all time points for the clusters generated by k-means clustering of differentially expressed genes between 4h of DMSO and 4h of ERKi. **(I)** Boxplot representation of the clusters generated in **(H)** for their expression at all time points. **(J)** Gene ontology categories representing the clusters generated in **(H)**.

Intrigued by the strong effect of ERK signalling on the phenotypic remodelling from an epithelial to mesenchymal state, we next sought to delineate the global transcriptional remodelling under the control of the ERK pathway. To this end we performed a genome-wide expression analysis and compared cells undergoing TGF-β-induced EMT with cells having compromised ERK signalling at 4h and 24h after EMT induction. A two-dimensional principal component analysis (PCA) was used to determine the variation between the samples (Figure 2A). Interestingly, as cells underwent EMT, their transcriptomes consecutively diverges on both principal components, whereas the ERK-inhibited cells exhibited changes in the second principal component that became more similar to the untreated condition. This result suggests that these are the genes leading to phenotypic reversal. The transcriptome analysis between cells undergoing normal EMT and cells with compromised ERK signalling indicated a significant fraction of deregulated genes at the early time point (4h; up=217, down=341), with more pronounced changes at the late time point (24h; up=551, down=590) (Figure 2B and 2C). Genes that exhibited expression level changes during EMT in normal cells but not in ERK-inhibited cells include several epithelial genes, such as Nid2, Cdkn1a, Id2, Tjp3, Crb3 and Wnt4 (Figure 2D and 2E), and key mesenchymal genes, such as Il11, Snai1, Hmga2, Ltbp1, Sox9 and Lamb1 (Figure 2F and 2G).

To comprehensively delineate which of the transcriptional changes induced during EMT (Figure 2, Supplementary Figure 2) are dependent on ERK signalling, we chose the genes that were differentially expressed between both conditions at 4h of EMT, analysed their expression at both time points and plotted a k-means (k=5) clustered heatmap representing their expression (Figure 2H). Interestingly, we discovered three clusters of genes (Cluster 1, 2 and 4) that exhibited significantly altered expression during EMT and showed a transcriptional reversal towards the untreated condition after ERK inhibition (Figure 2I and Supplementary Figure 1C); these genes likely explain the compromised potential to undergo EMT. Cluster 1 contained the genes that were downregulated during EMT but not after ERK signalling inhibition. Genes of this cluster were associated with gene ontology categories such as epithelium development and epithelial cell differentiation and included Shroom3, Id2, Gata5, Crb2 and Notch1 (Figure 2J cluster 1). Cluster 4 contained the genes that were highly induced in the early stages of EMT but were not activated when ERK was inhibited. These genes were associated with gene ontology categories such as the regulation of cell motility, migration, locomotion and metabolic processes (Figure 2J, cluster 4). They encode ligands, signalling molecules, receptors, chemotactic factors and integrins, such as Cx3cl1, Jak2, Tgfbr1, Ccl2 and Itga2, which are essential for the early cellular changes during EMT. Cluster 2 contained the genes that were continuously upregulated during EMT, exhibiting the highest expression levels at 24h but were not upregulated in cells that displayed compromised ERK signalling. These genes were also associated with the regulation of locomotion, cell motility and migration (Figure 2J, cluster 2) and include Hmga2, Il11, Sox9, Lamb1, Enc1, Itgb3, Jag1 and Col1a1, which are reported to be essential for the acquisition of a mesenchymal phenotype.

The remaining clusters did not show a transcriptional reversal in an ERK-dependent manner and the genes within these clusters did not associate with EMT-relevant GO-terms. Cluster 3 included genes that were strongly induced by ERK inhibition and were associated with GO terms such as inflammatory response, response to bacteria and regulation of cell death (Figure 2J, cluster 3), whereas cluster 5 comprised genes that were strongly downregulated in the ERK-inhibited condition and were associated with the regulation of MAPK signalling and numerous ribosome-associated GO terms (Figure 2J, Cluster 5). These two clusters probably represent secondary effects on gene expression as a result of the inhibitor treatment and most probably contain genes that are not directly related to EMT biology. For a comprehensive view of the transcriptional changes that occur during EMT and depend on ERK signalling, we performed the same analysis for the genes that were differentially expressed at 24h (Supplementary Figure 2-3). However, as shown by the derived clusters (Supplementary Figure 2A), the results did not show an increase in the essential informative clusters (cluster 2, 3 and 6) but rather seemed to capture more secondary effects that were independent of the transcriptional reversal towards an untreated fate (cluster 1, 4 and 5), although we did capture a few more essential genes that exhibited transcriptional reversal, such as Snai1, Mmp9 and Hnf4a. In summary, we identified a substantial set of EMT-relevant genes that display transcriptional induction or repression in an ERK-dependent manner during EMT, which is required for the phenotypic changes during this process.

### ERK signalling mediates global epigenetic reprogramming during EMT

**Figure 3:**
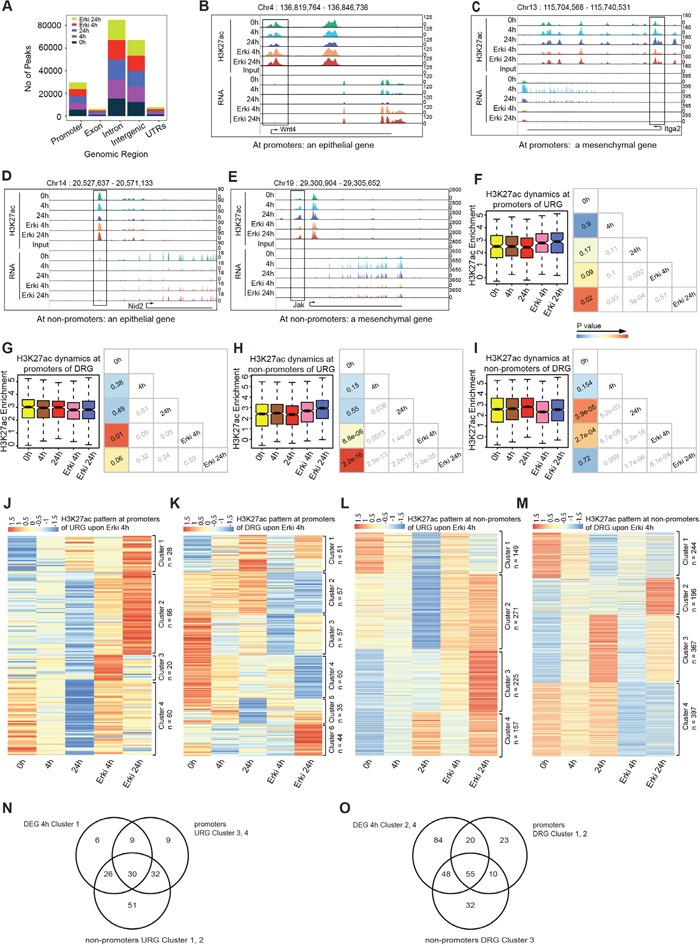
ERK pathway impacts upon H3K27ac dynamics during EMT **(A)** Bar plots represent the genome-wide peak distribution of the H3K27ac sites. **(B, D)** UCSC Genome Browser tracks representing H3K27ac enrichment and expression levels at promoters **(B)** and non-promoters **(D)** of epithelial marker genes in all conditions. **(C, E)** UCSC Genome Browser tracks representing H3K27ac enrichment and expression level at promoters **(C)** and non-promoters **(E)** of mesenchymal marker genes in all conditions. **(F)** Box plot represents the H3K27ac dynamics for the promoters of URGs with p-value cross correlation plot for each of these time points. **(G)** Box plot represents the H3K27ac dynamics for the promoters of DRGs with p-value cross correlation plot for each of these time points. **(H)** Box plot represents the H3K27ac dynamics for the non-promoters of URGs with p-value cross correlation plot for each of these time points. **(I)** Box plot represents the H3K27ac dynamics for the non-promoters of DRGs with p-value cross correlation plot for each of these time points. **(J)** Heatmap of H3K27ac dynamics at all time points at promoter regions of URG-representing clusters generated by k-means clustering. **(K)** Heatmap of H3K27ac dynamics at all time points at promoter regions of DRG-representing clusters generated by k-means clustering. **(L)** Heatmap of H3K27ac dynamics at all time points at non-promoter regions of URG-representing clusters generated by k-means clustering. **(M)** Heatmap of H3K27ac dynamics at all time points at non-promoter regions of DRG-representing clusters generated by k-means clustering. **(N)** Venn diagram representing the overlap of cluster 1 from 4h DEGs (Figure 2H) with clusters 3 and 4 from URG promoters and clusters 1 and 2 from URG non-promoters. **(O)** Venn diagram representing the overlap of clusters 2 and 4 from 4h DEGs (Figure 2H) with clusters 1 and 2 from DRG promoters and cluster 3 from DRG non-promoters. DRG: Downregulated genes in ERKi condition versus DMSO; URG: upregulated genes in ERKi condition versus DMSO; DEG: Differentially expressed genes in ERKi condition versus DMSO.

Intrigued by the ERK-dependent transcriptional reprogramming, we next investigated whether these changes can be explained by the influence of ERK on epigenetic remodelling during EMT. Active regulatory elements, such as promoters and enhancers are accessible and marked by acetylation of lysine 27 at Histone H3 (H3K27ac), thereby allowing to study the activity state of both proximal and distal regulatory elements [21–23]. We therefore performed, a genome-wide analysis using ChIP-seq for H3K27ac at different time points of TGF-β-induced EMT in normal and ERK-inhibited conditions. The genomic distribution confirmed the enrichment of the H3K27ac at promoters and potential enhancers, which are primarily situated in intergenic and intronic regions (Figure 3A). A comparative analysis of H3K27ac peaks did not show differences in the overall numbers of peaks (Figure 3A). We next examined local changes at specific sites. An inspection of tracks in the UCSC Genome Browser for the H3K27ac mark at promoter and non-promoter regions revealed that epithelial (Nid2 and Wnt4) and mesenchymal (Jak and Itga2) genes displayed ERK-dependent H3K27ac remodelling accompanying the transcriptional changes (Figure 3B–3E).

To further unravel the ERK-dependent H3K27ac dynamics that occur during normal and ERK-inhibited EMT and uncover whether chromatin changes might explain the observed gene expression changes, we further analysed the H3K27ac dynamics at promoters and distal regulatory sites that were differentially expressed between cells undergoing normal and ERK-inhibited EMT after 4h (Figure 3F–3M). Interestingly, we observed a strong effect of ERK inhibition on the overall H3K27ac levels at all regions, a result in agreement with the observed transcriptional response (Figure 3F–3I). To predict whether these ERK-dependent H3K27ac changes might explain the phenotypic reversal towards an untreated state after ERK inhibitor treatment, we performed k-means clustering to assess the dynamics of H3K27ac at promoters and non-promoter regions for the genes that were deregulated at 4h (Figure 3J–3M). Similarly to our observations for gene expression, various clusters were found in all conditions that exhibited reversed H3K27ac status along with the expression changes for which they were selected, thus suggesting that ERK signalling is required for H3K27ac remodelling during EMT and thereby partially explains the ERK-dependent transcriptome remodelling. By analysing the promoters of genes that were upregulated at 4h of ERK-inhibited EMT induction, we found that clusters 3 and 4 exhibited decreased H3K27ac levels during normal EMT but reverted towards levels observed in epithelial cells in the ERK-inhibited condition. These clusters contained epithelial genes, such as Wnt4, Id3 and Cldn9 (Figure 3J). Moreover, in the analysis of the promoters of genes that were downregulated in ERK-inhibited EMT at 4h, the genes in clusters 1 and 2 displayed increased H3K27ac levels during normal EMT but not after ERK inhibition (Figure 3K). Strikingly, these clusters contained EMT-relevant genes, such as Hmga2, Il11, Itga2, Tgfbr1 and Lamb1, which also exhibited transcriptional reversal (Supplementary Figure 4A).

Because the number of EMT-relevant differentially expressed genes that could be explained by an ERK-dependent H3K27ac dynamic at promoters was limited, we explored whether distal H3K27ac sites might regulate the remaining set of EMT-relevant changes that occur after ERK inhibition. In this analysis, at non-promoter regions of genes that were upregulated after ERK inhibition at 4h, we found that clusters 1 and 2 (n=149 and n=271 regions, respectively) exhibited decreased H3K27ac levels during normal EMT but not after ERK inhibition (Figure 3L). These potential enhancer elements that exhibited a more epithelial-like H3K27ac level after ERK inhibition were associated with genes that are critical for epithelial biology, such as Cmtm8, Wnt4 and Shroom3. Moreover, these genes also showed expression level reversal after ERK inhibition (Supplementary Figure 4B) and might therefore be regulated by distal regulatory regions. The genes nearest to these potential enhancers were strongly associated with signalling pathways and apoptosis as well as with tissue development, response to wounding, positive regulation of cell communication and epithelial cell development, thus suggesting their importance in epithelial biology (Supplementary Figure 4C). An inspection of H3K27ac kinetics at non-promoter regulatory regions of genes downregulated at 4h of ERK-inhibited EMT revealed that cluster 3 (n=367 regions) exhibited substantial H3K27ac enrichment during normal EMT but reverted towards epithelial status in the ERK-inhibited condition (Figure 3M). These potential enhancers were located close to mesenchymal state-promoting genes, such as Il11, Hmga2, Lamb1, Jag1 and Itgb3 (Supplementary Figure 4D), which also exhibited downregulated expression levels after ERK inhibition. GO terms for these genes were associated with many signalling cascades, including the MAPK cascade and EMT-relevant processes, such as cell migration, locomotion, gastrulation and cell motility (Supplementary Figure 4E), thus suggesting the potential for ERK-dependent enhancer activation, which leads to a gain in H3K27ac status during EMT. Having observed gene clusters that were essential for maintaining epithelial cell-like expression patterns in ERK-inhibited EMT (Figure 2H) and might explain the failure of cells to undergo proper EMT, we sought to determine the proportion of these genes whose expression changes could be explained by H3K27ac patterns either at promoters or potential enhancer elements. For the genes that were essential for epithelial biology and downregulated during normal EMT but not when ERK signalling was compromised (Figure 2H, cluster 1), almost all genes exhibited a reverted H3K27ac pattern either at promoters (~55%), potential enhancers (~78%) or both (~42%), thus suggesting that ERK-dependent transcriptional remodelling is consistent with epigenetic changes (Figure 3N). Similarly, more than half (60%) of the mesenchymal genes that were transcriptionally induced during EMT in an ERK-dependent manner (Figure 2H, cluster 2 and 4) also showed a reverted H3K27ac pattern at promoters (~36%), enhancers (~50%) or both (~27%) (Figure 3O).

### ERK-dependent epigenetic reprogramming contributes to transcriptional changes during EMT

**Figure 4:**
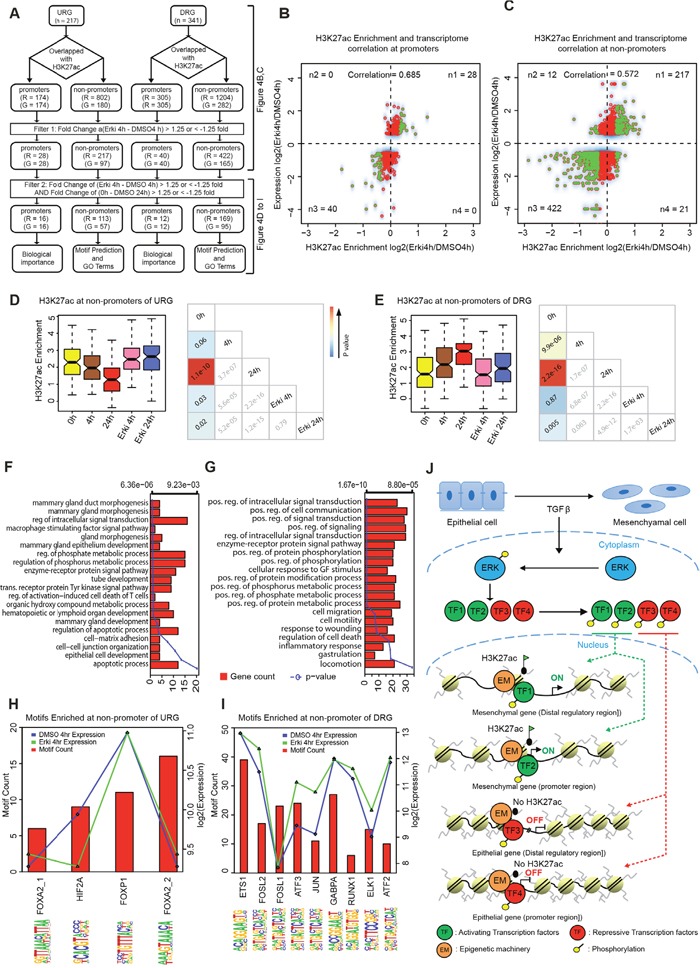
ERK-dependent chromatin changes contribute to transcriptional reprogramming during EMT **(A)** Scheme representing the strategy for downstream analysis. **(B)** Correlation of H3K27ac dynamics and gene expression at promoter regions (n1, n2, n3, n4 represent the number of significant H3K27ac sites at the respective gene). **(C)** Correlation of H3K27ac dynamics and expression of the nearest gene at non-promoter regions (n1, n2, n3, n4 represents the number of significant H3K27ac sites at differentially expressed genes). **(D)** Boxplot representing the significantly regulated non-promoters sites of URG in 1) the normal EMT time course between 0h and 24h and 2) between the 4h time points of ERKi and DMSO, with p-value cross correlation plots for each of these conditions below. **(E)** Boxplot representing the significantly regulated non-promoters sites of DRG in 1) the normal EMT time course between 0h and 24h and 2) between the 4h time points of ERKi and DMSO, with p-value cross correlation plots for each of these conditions below. **(F)** GO terms for the URGs that significantly changes 1) between ERKi 4h and DMSO 4h and 2) in the normal EMT time course between 0h and 24h. **(G)** GO terms for the DRGs that significantly changes 1) between ERKi 4h and DMSO 4h and 2) in the normal EMT time course between 0h and 24h DMSO. **(H)** Plot representing the motifs enriched at non-promoter sites of URGs. **(I)** Plot representing the motifs enriched at non-promoter sites DRGs. **(J)** Model showing ERK dependent epigenetic reprogramming during EMT: During TGF-β induced EMT, ERK signalling becomes activated and in turn phosphorylates downstream effectors including transcription factors (TFs) that translocate into the nucleus and establish gene expression program required for EMT. Such activating and repressing transcription factors interact with members of the epigenetic machinery (EM) to set up chromatin landscape changes at promoters and enhancer elements to activate mesenchymal and repress epithelial genes.

Since ERK signalling was found to be essential for a subset of the transcriptional and epigenetic changes that accompany normal EMT, we performed a more comprehensive investigation of the association between the H3K27ac and transcriptome changes during EMT and their dependence on ERK signalling (Figure 4A). Thus, we examined the global H3K27ac changes between normal and ERK-inhibited EMT at 4h in promoter and non-promoter regions at genes that were differentially expressed under these conditions (Figure 4B and 4C, Supplementary Figure 5A). Strikingly, we observed a very strong correlation at promoters and non-promoters, thus suggesting that ERK-dependent transcriptional remodelling is linked to H3K27ac dynamics. Whereas a small set of genes displayed ERK-dependent regulation of H3K27ac at their promoters (number of genes (G) = 68), a far larger set of genes (G=262) was regulated by H3K27ac changes at potential enhancer regions (number of regions (R) =639), thus suggesting that ERK signalling affects gene regulation via enhancer regulation. To extract the subset of such regions that might explain the observed epithelial phenotype of cells during ERK-inhibited TGF-β-induced EMT, we selected the set of regions that exhibited significantly induced or suppressed H3K27ac levels during normal EMT and reverted in both H3K27ac and expression towards an epithelial state in ERK-compromised EMT (Figure 4A). Strikingly, even though these regions were selected from the 4h time point, the reversal was equally strong and highly significant at both 4h and 24h (Figure 4D and 4E, Supplementary Figure 5B and 5C). This finding clearly indicates that such regions are important not only at the onset of EMT but also at later time points, when the cells are in a more mesenchymal state. The gene ontology categories that were enriched for the genes that lost expression and H3K27ac levels at their nearest potential enhancer during EMT in an ERK-dependent fashion relate to epithelial cell development, mammary gland epithelial development, several signalling and metabolic processes (Figure 4F). These results suggest that these genes are important in epithelial biology and the tuning of signalling and metabolism during cell fate change. The genes that gained expression and enhancer H3K27ac levels in an ERK-dependent manner during EMT were enriched in GO terms associated with cell migration, motility, locomotion and wound healing, thus indicating their involvement in the acquisition of a mesenchymal, motile phenotype. In addition, several metabolic and signalling GO terms were also enriched that again relate to different requirements during cell fate changes (Figure 4G).

Next, we sought to predict transcription factors that may function at distal genomic sites that are epigenetically remodelled during EMT and contribute to gene expression changes. Therefore, we performed a motif prediction at the distal regions that exhibited reverted H3K27ac levels and examined the expression of the predicted binding factors during normal EMT (Figure 4H–4I and Supplementary Figure 5D-5E). This analysis revealed the genes that were downregulated during EMT in an ERK-dependent fashion might be directly acted upon by FOXA2, HIF2A and FOXP1, all of which are expressed at the relevant time point at 4h of EMT (Figure 4H and Supplementary Figure 5D). For the genes that were upregulated during EMT in an ERK-dependent manner, we identified ETS1, FOSL2, FOSL1, ATF3, JUN, GABPA, RUNX1, ELK1 and ATF2, which were also all expressed at the relevant EMT time point, thus suggesting that they are potential downstream factors of ERK signalling that function at these regions (Figure 4I and Supplementary Figure 5E). Overall, we identified a distinct set of EMT-relevant genes that undergo epigenetic and transcriptional reprogramming in an ERK-dependent manner and further predicted the downstream effectors that may mediate these responses.

## DISCUSSION

Epithelial to mesenchymal transition allows cells to migrate and therefore, it is a key process during development, wound healing, fibrosis and cancer metastasis. The phenotypic remodelling can be initiated by several signalling molecules that activate a plethora of downstream signalling cascades. A prototypic inducer of EMT is TGF-β, which is known to activate both the canonical Smad signalling pathway and several non-canonical pathways, including MAPK signalling. Although the interplay of several pathways has become increasingly clear and key EMT genes, such as Snail Twist and Zeb1 have been identified, the transcriptional networks induced by each pathway remain poorly understood. Here, we delineated the global transcriptional response during TGF-β-induced EMT in a model system of mouse mammary gland epithelial cells (NMuMG) and further narrowed the fraction of transcriptional responses that are dependent on ERK signalling by using an established ERK inhibitor. Strikingly, we observed that cells inhibited for ERK signalling shortly before TGF-β induction failed to undergo proper EMT and are retained in an epithelial state as further reflected by the phenotype and transcriptome analysis. Our results suggest that ERK signalling is critical for the onset of EMT given its induction at early stages of TGF-β induced EMT and observations that its inhibition compromises molecular and phenotypic changes required for acquiring mesenchymal identity. In light to our earlier findings where we showed that JNK signalling is inducted at later time points during EMT and is essential for progression of EMT but not its onset [20]. Such differences in induction kinetics of several EMT-relevant signalling pathways vouches for a comprehensive analysis to decipher how such networks collaboratively induce consecutive steps of TGF-β induced EMT and how this involves coordinated regulation of gene regulatory programs underlying EMT.

ERK inhibition led to failure of cells to induce critical EMT genes, such as Il11, Snai1, Hmga2, Sox9 and Lamb1. Moreover, the downregulation of key epithelial genes, such as Cdkn1a, Id2, Tjp3, Crb3 and Wnt4, was compromised, thus suggesting that active ERK signalling is essential for such transcriptional responses driving EMT. Interestingly, we also observed that the transcriptional response in ERK-inhibited cells at the late time point (24h) was more similar to that of the untreated condition (0h). We further examined the transcriptional dynamics during EMT and the dependence of early- versus late-induced transcriptional changes on ERK signalling. Interestingly, we clearly identified a set of ERK-dependent genes that were most strongly induced at 4h of EMT and were not highly activated at later time points. These genes had functions in locomotion and cell motility but displayed an overrepresentation of metabolic processes and signalling cascades, thus suggesting that the early changes strongly regulate the altered metabolic needs of cells that migrate out of connected tissues and the induction of secondary signalling cascades that transmit the original signal. The class of genes strongly induced at later EMT time points (24h) did not show such overrepresentation of metabolic and signalling processes but were mostly related to motility, migration, locomotion and movement, thus supporting the acquisition of a motile mesenchymal phenotype. Overall, the most EMT-relevant transcriptome changes were observed at the early time point (4h), while the later time point (24h) also included genes associated with cell cycle regulation as well as DNA metabolic processes in addition to few EMT relevant genes. This may simply reflect an accumulation of primary and secondary effects at later time points due to a prolonged inhibition of ERK signalling, where we may also start to see influence of inhibiting its known function in cellular proliferation [24] [25] [26].

A body of emerging evidence suggest that the fast integration of extracellular cues is essentially achieved by signalling cascades directly affecting chromatin and thereby ensuring a fast and precise transcriptional response [10]. Although the importance of epigenetic remodelling during cell fate changes is well known, genome-wide studies for chromatin alterations during EMT are sparse, and they have not focused on the effects of one particular signalling pathway on epigenomic changes [11]. Therefore, we chose to analyse the ERK signalling-dependent remodelling of the active chromatin mark H3K27ac, which is present at active promoters and enhancers, at different time points during TGF-β-induced EMT. Strikingly, we found that a large proportion of EMT-relevant genes that were transcriptionally regulated during EMT in an ERK-dependent manner associated with promoters or enhancer elements that displayed ERK-dependent remodelling of this chromatin mark. Therefore, we were able to show that a significant fraction of H3K27ac sites at promoters and intergenic regions become remodelled partly in an ERK-dependent fashion during EMT. This study thus provides the first description of an enhancer landscape that is remodelled in an ERK dependent manner during EMT and regulates the silencing of epithelial genes, such as Wnt4, Cmtm8, Shroom3 and the activation of mesenchymal genes, such as Hmga2, Il11, Itga2, Tgfbr1 and Lamb1. While these genes we exemplified have been established to be important for epithelial or mesenchymal identity, our study uncovers the regulatory elements that potentially function to control their expression and further offers a basis for investigation into the molecular players operating at these regions in their transcriptional regulation.

Since previous studies in mouse and human embryonic stem cells [16] demonstrated direct binding of ERK to chromatin [17, 18] we had also tested whether ERK directly binds chromatin during different stages of EMT. However, our attempts to do ChIP-seq assay using several endogenous antibodies as well as following overexpression of tagged versions of ERK1 and ERK2 during EMT showed no reliable binding sites (data not shown). Therefore, at least in the context of EMT, ERK does not function by a direct binding to chromatin and may exclusively employ downstream factors to mediate epigenetic reprogramming. To predict such factors we performed motif analysis at genomic regions losing and gaining H3K27ac that indicated the enrichment of a distinct set of transcription factors, many of which are known to be involved in EMT [27–30]. These factors offer ideal candidates for further mechanistic investigation of their function downstream of ERK in driving the observed epigenetic and transcriptional responses. To extend these findings, future work should also involve generating a phospho-proteome with and without ERK inhibition at early EMT time points to unravel transcription factors that are phosphorylated in an ERK-dependent manner during onset of EMT.

Overall, our data provide the first delineation of early and late transcriptional responses during TGF-β-induced EMT that rely on ERK signalling and relate these changes to the epigenetic reprogramming of the active regulatory landscape. These data further uncover distinct transcription factors that function downstream of ERK and cooperate with epigenetic machinery to mediate epigenetic reprogramming at regulatory elements of EMT-relevant genes (Figure 4J). Given these evidences for ERK signalling mediated chromatin remodelling to drive transcriptional program underlying EMT, it is desirable to gain a comprehensive understanding of the involved players and kinetics of these processes. Furthermore, it would be essential to validate the generality of our findings to different contexts of EMT such as gastrulation [31, 32], neural crest formation [33], tissue regeneration and wound healing [3, 4], as well as pathogenic conditions such as fibrosis [5] and metastasis formation during tumorigenesis [6] [34]. Deciphering these mechanisms will allow us to uncover the basic principles of gene regulation driving cell-fate changes, at the same time offer possibility to reveal novel therapeutic avenues for the treatment of disorders involving aberrant EMT, such as cancer.

## MATERIALS AND METHODS

### Cell culture

For our study we used a subclone of NMuMG cells (NMuMG/E9, hereafter NMuMG) that has been described previously [19] and was grown in DMEM supplemented with 10% FBS, 2 mM L-Glutamine and 1X Non-essential amino acids and cultured at 37°C with 7% CO_2_ in a humid incubator. For EMT induction, NMuMG cells were treated with 2 ng/ml TGF-β (rhTGF-β1 240-B, R&D systems) for the indicated time points.

### Inhibitor treatment

Cells were seeded at same densities and pretreated 30 min prior to TGF-β treatment with ERK-signaling inhibitors UO126 (25μM) and PD98095 (50μM) or their solvent DMSO and were subjected to respective experiments and analyzed at indicated time points.

### Immunofluorescence assay

NMuMG cells were grown on coverslips, fixed with 4% paraformaldehyde in PBS and permeabilized with 0.2% Triton X-100 for 15 minutes at room temperature. Subsequently, cells were blocked for 20 minutes at room temperature in blocking solution (10% goat serum, 5% FCS and 0.5% BSA in PBS) followed by incubation with primary antibodies at 4°C overnight. These samples were washed three times with PBS for 10 minutes at room temperature following incubation with fluorochrome-labeled secondary antibody or Phalloidin-633 as well as Hoechst for DNA counterstain (20ng/ml) for 1 hour at room temperature. For mounting of the samples Immu-Mount (Thermo Scientific) reagent was used and samples were imaged with a confocal laser-scanning microscope (SP05, Leica). Data were processed with ImageJ software.

Antibodies used for immunofluorescence were E-Cadherin (13-1900, Invitrogen and 610182, BDTransduction Laboratories), N-Cadherin (610921, BD Transduction Laboratories), ZO-1 (617300, Invitrogen), Alexa Fluor-488 goat anti mouse IgG (H+L) (A11029, Invitrogen); Alexa Fluor-568 goat anti rabbit IgG (H+L) A11011 Invitrogen; and Alexa Fluor-633 goat anti rat IgG (H+L) A21094, Invitrogen, Alexa Fluor 633 Phalloidin (A22284, Invitrogen) was used to stain F-actin.

### RNA extraction

RNA was extracted using the SurePrep TrueTotal RNA Purification Kit (Fisher Scientific) according to manufactures instructions.

### Immunoblotting

Cells were lysed in RIPA buffer (50mM Tris (pH8), 1%NP40, 0.5% sodium deoxycholate, 0.1%SDS, 150mM NaCl, 5mM EDTA, 1% Glycerol, 2.5mM MgCl, 2mM sodium orthovanadate) and protein concentrations were quantified by using Bradford reagent (Biorad). Equal amounts of proteins (30 μg) were boiled in 6 × SDS–PAGE loading buffer and run on a polyacrylamide gel and transferred to a PVDF membrane and probed with respective antibodies.

Antibodies used for immunoblotting were Phospho-p44/42 MAPK (Erk1/2) (Thr202/Tyr204) Antibody #9101, Erk1/2 (sc94, santa cruz) and GAPDH (221-MG-GAPDH, Immuno Kontact).

### ChIP assay

NMuMG cells were cross-linked in growth medium containing 1% formaldehyde for 10 min at room temperature, neutralized with 0.125M glycine, scraped off and rinsed twice with 10 ml 1X PBS. The fixed cells were subjected to preparation of nuclear lysates. In detail pellets were resuspended in 3 mL of Buffer L1 (50mM Hepes KOH, pH 7.5, 140mM NaCl, 1mM EDTA pH 8.0, 10% glycerol, 5% NP-40, 0.25% Triton-X 100) and incubated for 10 min at 4°C. This was followed by centrifugation for 5 min at 4°C at 1300g. The pellet was then resuspended in 3 ml of Buffer L2 (200mM NaCl, 1mM EDTA pH 8.0, 0.5mM EGTA pH 8.0, 10mM Tris pH 8.0) and incubated for 10 min at room temperature, followed by centrifugation for 5 min at 4°C at 1300g. The pellet was then resuspended in 600 μl Buffer L3 (1mM EDTA pH 8.0, 0.5mM EGTA pH 8.0, 10mM Tris pH 8.0, 100mM NaCl, 0.1% Na-deoxycholate, 0.17mM N-Lauroyl sarcosine) containing protease inhibitors and incubated at 4°C for 3 hours following sonication using Bioruptor plus (Diagenode). 60 μg of chromatin was incubated overnight at 4 °C with 2 μg of the antibody targeting H3K27ac (ab4729; Abcam) to allow formation of the immunocomplex. To pull down H3K27ac enriched DNA fragments the mixture was then incubated for 3 h with 40 μl protein A-Sepharose beads preblocked with tRNA and BSA at 4°C. To reduce background signal these beads were subjected to several washing steps employing different buffers. For this beads were washed twice with 1 ml buffer L3 and once with 1 ml DOC buffer (10 mM Tris (pH 8.0), 0.25 M LiCl, 0.5% NP-40, 0.5% deoxycholate, 1 mM EDTA) and transferred to a fresh Eppendorf tube using TE buffer. The bound chromatin was eluted in 1% SDS/0.1 M NaHCO_3_ in two rounds for 20 minutes and eluates were combined. The eluate containing the immunocomplex bound to the precipitated DNA was treated with RNase A (0.2mg/ml) for 30 min at 37°C and then with proteinase K (50 μg/ml) for 2.5 h at 55°C. The crosslinking was reversed at 65°C overnight with gentle shaking and precipitated DNA was purified by phenol-chloroform extraction followed by ethanol precipitation and recovered in 40 μl TE buffer.

### RNAseq data analysis

RNA sequencing for untreated (0hr), 4hr, 24hr time points under DMSO and ERK inhibition conditions were performed. After receiving RNA sequencing output in fastq format, quality check was performed using FASTQC version v0.10.0 followed by alignment using TopHat version v2.0.8 [35] to the mouse genome (mm9) with default parameters. Mapping reads were considered for read count per gene using HTSeq version 0.5.4p1 [36]. Output from HTSeq (Read counts) were normalized and differential gene expression analysis was performed using a R package DESeq with FDR rate of 0.1 [37]. Differential expression analysis between 4hr time point from DMSO and ERK inhibited conditions were performed. Similar analysis was also performed for 24hr time point. GO (Gene Ontology) analysis of the Differentially expressed genes was performed using ToppGene [38].

### H3K27ac ChIP-seq analysis

H3K27ac ChIP-seq was performed for the 0hr, 4hr and 24hr time points under the DMSO and ERK inhibition conditions. After quality check using FASTQC version v0.10.0 sequencing reads were aligned to reference mouse genome (mm9) using bowtie version 0.12.9 [39] with default parameters. Output obtained after alignment (.sam) converted to. bam using samtools version: 0.1.19-44428cd [40]. MACS2 version 2.0.10.20120913 [41] was used for peak calling with default parameters. Called peaks from each samples containing replicates were merged together for the peak reproducibility. Raw enrichment for each merged sample with respect to input was calculated by using bam file from replicates for each condition using QuasR package [42]. Further, Normalized Enrichment with respect to input for each of the condition was calculated. Peak displaying 1.5 fold enrichment above input were selected for subsequent analysis. All the significantly enriched peaks were categorized into promoter and non-promoter peaks. The peaks regulating the differentially regulated genes at 4hr conditions were screened and used for the further analysis.

### Browser track generation

Wig files were created for all the samples using QuasR package [42] and visualized using UCSC genome browser.

### Genome wide peak distribution

The gene annotation file for mouse (mm9) from UCSC was used and the peaks were annotated using HOMER utility [43] annotatePeaks.pl, which gives the information of the number of peaks present at the promoter, intergenic regions, exon, intron and UTRs.

### Motif analysis

Motif Analysis was performed for distal regulatory elements using Homer utility [43] on the regions significantly regulated during 4hr conditions (i.e. 4hr DMSO and 4hr ERK inhibition) and untreated to 24hr DMSO condition by 1.25 fold.

## SUPPLEMENTARY MATERIALS FIGURES AND TABLES


