# Supplementary material for: ERK signalling modulates epigenome to drive epithelial to mesenchymal transition
Source: Oncotarget. 2017 Mar 23;8(17):29269–81. doi: 10.18632/oncotarget.16493 (PMC5438729; doi:10.18632/oncotarget.16493)
Supplement: Supplementary file 1 [file oncotarget-08-29269-s001.pdf]

ERK signalling modulates epigenome to drive epithelial to mesenchymal transition

SUPPLEMENTARY FIGURES

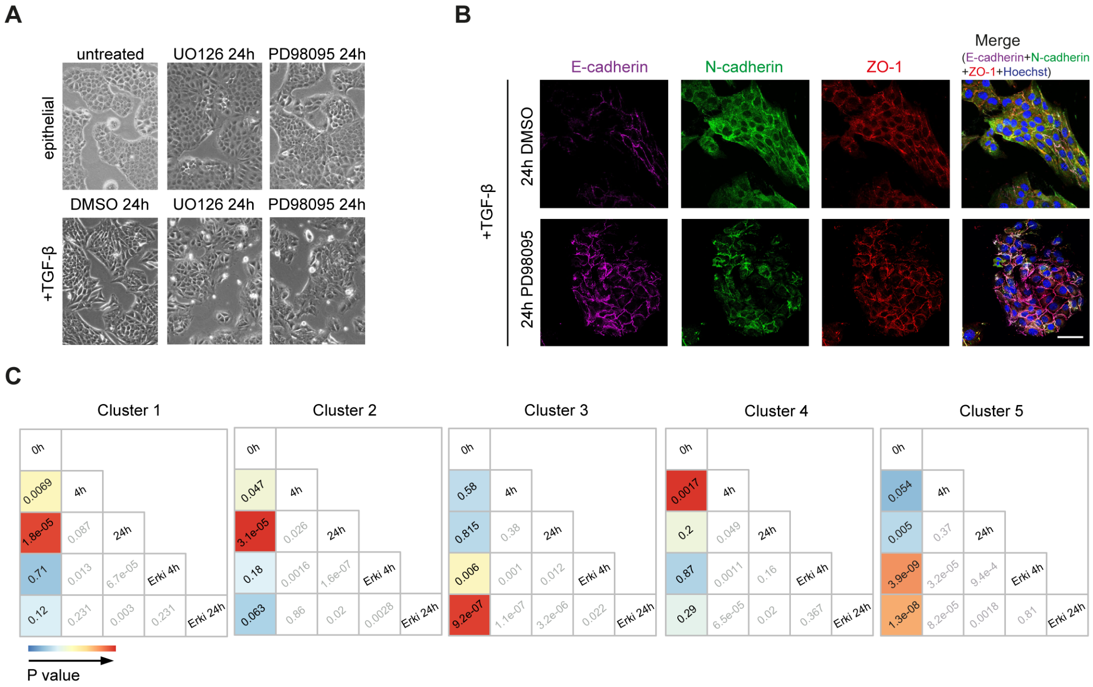

Supplementary Figure 1: (A) Phase contrast images of untreated NMuMG cells and cells treated for 24h with TGF-β in the presence (UO126; PD98095) or absence (DMSO) of the ERK inhibitor. **(B)** Immunofluorescence microscopy analysis of changes in the localization and expression levels of marker proteins in cells treated with TGF-β in the absence (DMSO) and presence (PD98095) of a different ERK pathway inhibitor. The staining was performed with antibodies against the epithelial markers E-cadherin and ZO-1 and the mesenchymal markers N-cadherin. Scale bar, 50 µm, 63X magnification. **(C)** p-value cross correlation plots for each of the clusters obtained in 2H and shown as box plots 2I at all time points.

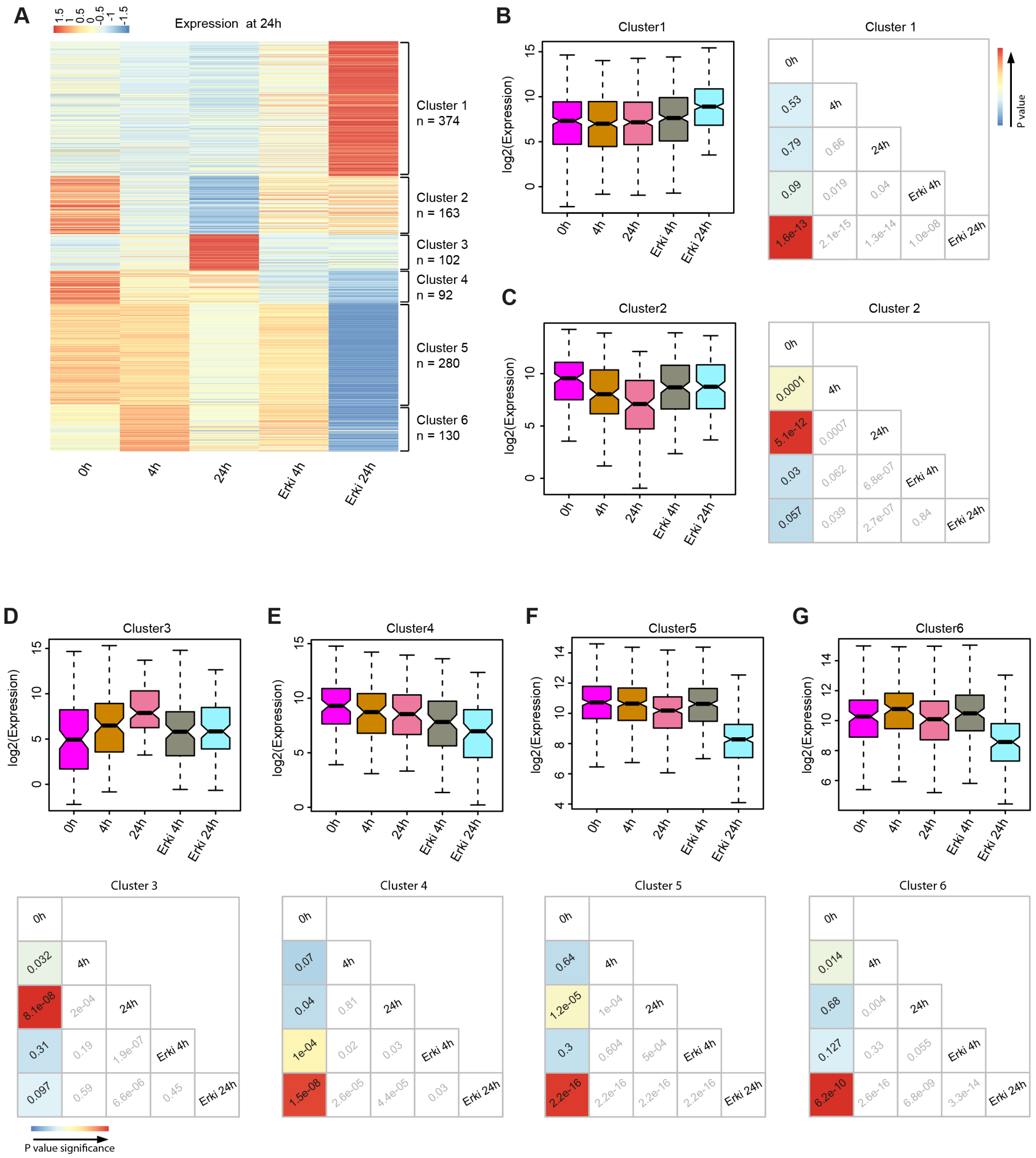

Supplementary Figure 2: (A) Heatmap representation at all time points for the clusters generated by k-means clustering of differentially expressed genes between 24h DMSO and 24h ERKi. **(B-G)** Boxplot representation of the clusters generated in **(A)** for their expression at all the time points, with p-value cross correlation plots for each of these clusters at all time points.

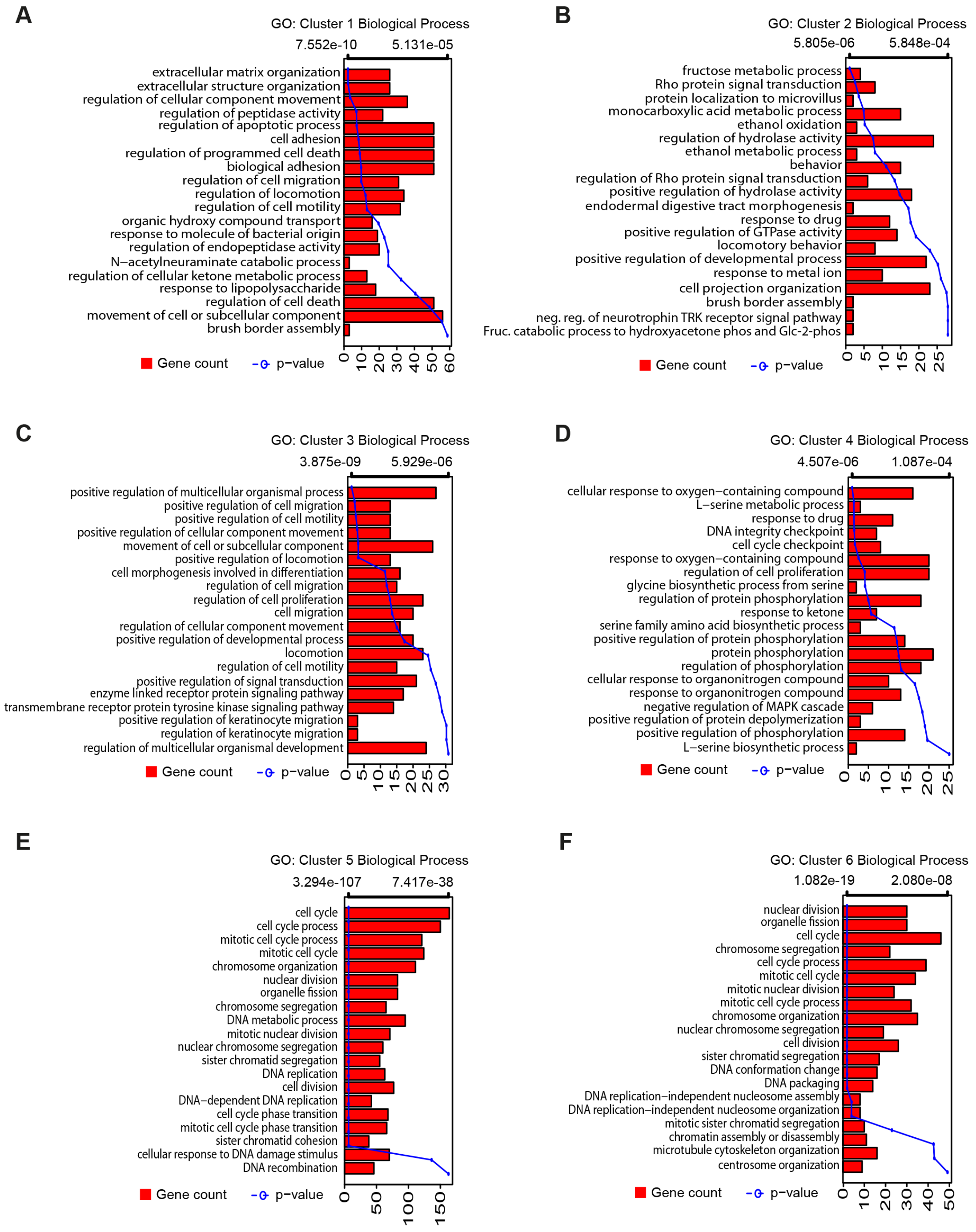

Supplementary Figure 3: GO terms for the transcriptional changes at 24h EMT and ERKi EMT: (A) Gene ontology categories for cluster 1 from Supplementary Figure 2A. **(B)** Gene ontology categories for cluster 2 from Supplementary Figure 2A. **(C)** Gene ontology categories for cluster 3 from Supplementary Figure 2A. **(D)** Gene ontology categories for cluster 4 from Supplementary Figure 2A. **(E)** Gene ontology categories for cluster 5 from Supplementary Figure 2A. **(F)** Gene ontology categories for cluster 6 from Supplementary Figure 2A.

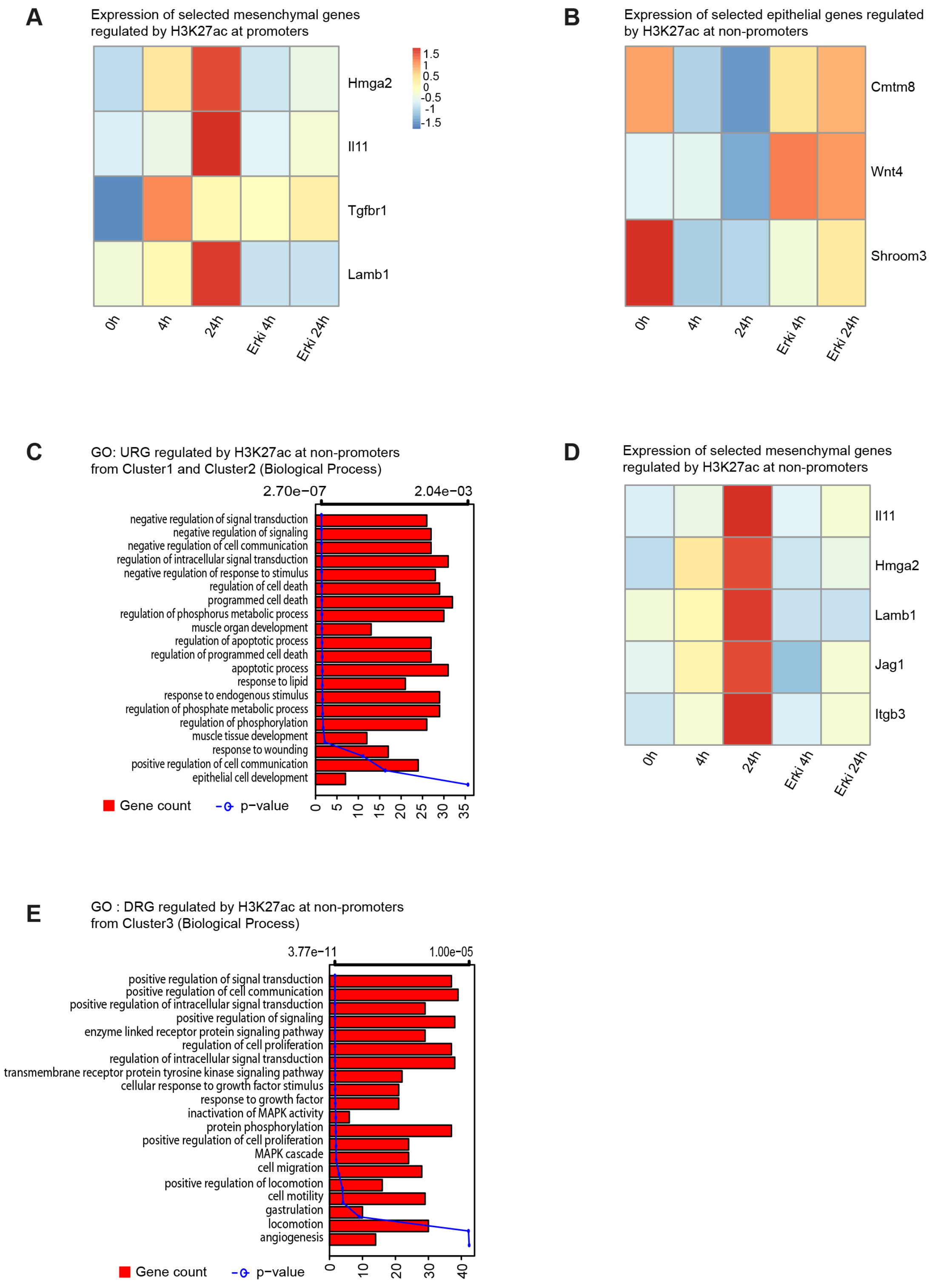

Supplementary Figure 4: (A) Heatmap representing expression level of selected key mesenchymal genes regulated by H3K27ac at promoter regions. **(B)** Heatmap representing expression level of selected epithelial genes regulated by H3K27ac at non-promoter regions. **(C)** GO term representation for the URGs regulated by H3K27ac mark at non-promoter regions (cluster 1 and cluster 2 from Figure 3L) **(D)** Heatmap representing expression level of selected mesenchymal genes regulated by H3K27ac at non-promoter regions. **(E)** GO term representation for the DRGs regulated by H3K27ac mark at non-promoter regions (cluster 3 from Figure 3M).

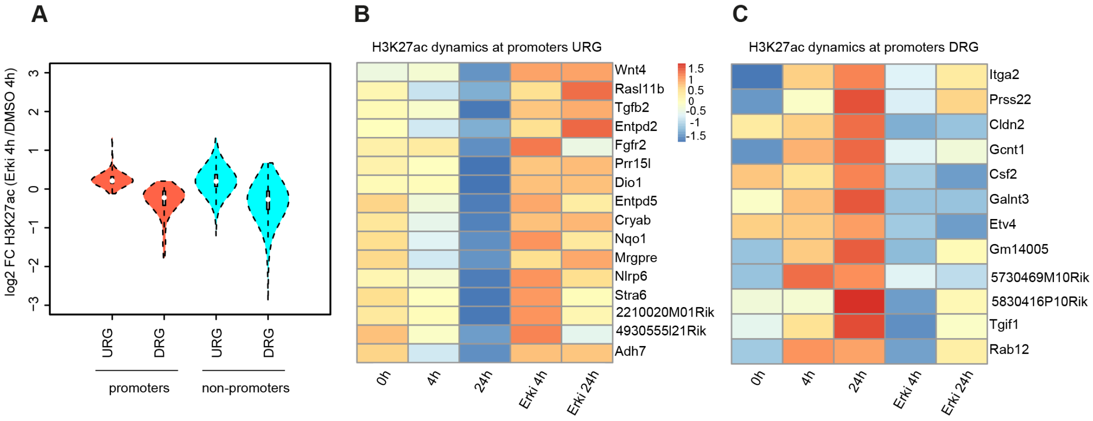

D Motifs Enriched at non-promoters sites of URG

Motif Name	P- value	No. of target Sequences	% of target sequences	% of background sequences	Fold change = target / background	Motif Logo
FOXA2_1	1.0e-03	6	5.31	0.95	5.58	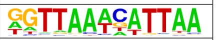
HIF2A	1.0e-02	9	7.96	2.5	3.18	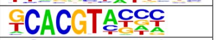
FOXP1	1.0e-02	11	9.73	3.72	2.61	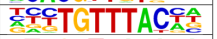
FOXA2_2	1.0e-02	16	14.16	7.36	1.92	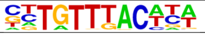

E Motifs Enriched at non-promoters sites of DRG

Motif Name	P- value	No. of target Sequences	% of target sequences	% of background sequences	Fold change = target / background	Motif Logo
ETS1	1.0e-05	39	23.08	10.42	2.21	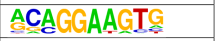
FOSL2	1.0e-05	17	10.06	2.78	3.61	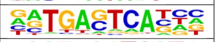
FOSL1	1.0e-05	23	13.61	4.76	2.85	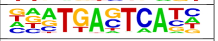
ATF3	1.0e-04	24	14.20	5.9	2.40	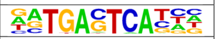
JUN	1.0e-03	11	6.51	2.03	3.20	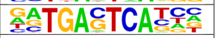
GABPA	1.0e-02	27	15.98	8.55	1.86	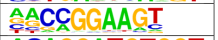
RUNX1	1.0e-02	06	3.55	0.73	4.86	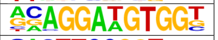
ELK1	1.0e-02	15	8.88	3.97	2.23	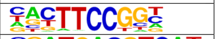
ATF2	1.0e-02	10	5.92	2.46	2.40	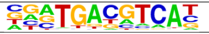

Supplementary Figure 5: (A) Violin plots representing the cut-off for the selection of the significantly regulated DEGs by H3K27ac at promoter and non-promoter in the comparison of the 4h ERKi and DMSO conditions. **(B)** Heatmap representation of the H3K27ac enrichment at all time points at the promoters of URGs, which were significantly regulated 1) between 4h ERKi and 4h DMSO and 2) in the normal EMT time course between 0h and 24h. **(C)** Heatmap representation of the H3K27ac enrichment at all time points at promoters of DRGs, which were significantly regulated 1) between 4h ERKi and 4h DMSO and 2) in the normal EMT time course between 0h and 24h. **(D)** List of the motifs enriched at non-promoters of URG. **(E)** List of the motifs enriched at non-promoters of DRG.
